# Perception of Thermal Pain and the Thermal Grill Illusion Is Associated with Polymorphisms in the Serotonin Transporter Gene

**DOI:** 10.1371/journal.pone.0017752

**Published:** 2011-03-15

**Authors:** Fredrik Lindstedt, Tina B. Lonsdorf, Martin Schalling, Eva Kosek, Martin Ingvar

**Affiliations:** 1 Osher Center for Integrative Medicine, Stockholm Brain Institute, Department of Clinical Neuroscience Karolinska Institutet, Stockholm, Sweden; 2 Department of Systems Neuroscience, University Hospital Eppendorf, Hamburg, Germany; 3 Neurogenetics Unit, Department of Molecular Medicine and Surgery and Center for Molecular Medicine, Karolinska Institutet, Stockholm, Sweden; Biological Research Center of the Hungarian Academy of Sciences, Hungary

## Abstract

**Aim:**

The main aim of this study was to assess if the perception of thermal pain thresholds is associated with genetically inferred levels of expression of the 5-HT transporter (5-HTT). Additionally, the perception of the so-called thermal grill illusion (TGI) was assessed. Forty-four healthy individuals (27 females, 17 males) were selected a-priori based on their 5-HTTLPR/rs25531 (‘tri-allelic 5-HTTLPR’) genotype, with inferred high or low 5-HTT expression. Thresholds for heat- and cold-pain were determined along with the sensory and affective dimensions of the TGI.

**Results:**

Thresholds to heat- and cold-pain correlated strongly (rho  = −0.58, p<0.001). Individuals in the low 5-HTT-expressing group were significantly less sensitive to heat-pain (p = 0.02) and cold-pain (p = 0.03), compared to the high-expressing group. A significant gender-by-genotype interaction also emerged for cold-pain perception (p = 0.02); low 5-HTT-expressing females were less sensitive. The TGI was rated as significantly more unpleasant (affective-motivational dimension) than painful (sensory-discriminatory dimension), (p<0.001). Females in the low 5-HTT expressing group rated the TGI as significantly less unpleasant than high 5-HTT expressing females (p<0.05), with no such differences among men.

**Conclusion/Significance:**

We demonstrate an association between inferred low 5-HTT expression and elevated thresholds to thermal pain in healthy non-depressed individuals. Despite the fact that reduced 5-HTT expression is a risk factor for chronic pain we found it to be related to hypoalgesia for threshold thermal pain. Low 5-HTT expression is, however, also a risk factor for depression where thermal insensitivity is often seen. Our results may thus contribute to a better understanding of the molecular underpinnings of such paradoxical hypoalgesia. The results point to a differential regulation of thermoafferent-information along the neuraxis on the basis of 5-HTT expression and gender. The TGI, suggested to rely on the central integration of thermoafferent-information, may prove a valuable tool in probing the affective-motivational dimension of these putative mechanisms.

## Introduction

The experience of pain and emotion are intertwined [1].Clinically, disorders involving the latter are often accompanied by reports of pain [2]. Equally, patients with chronic pain often suffer from affective disorders although the chain of causality linking the two remains to be established [3]. High frequencies of affective disorders, e.g. 30–60%, have been reported in various studies of patients with generalized pain [4] and, reciprocally, pain complaints in patients suffering from major depression appear to be extremely common [5]. In light of this close relationship, the view of pain as a homeostatic emotion seems especially apt [6].

Given the outlined co-morbidity between pain and affective disorders, one might expect that sensitivity to experimental pain would be increased in depressed patients. This is not always the case, however, and - paradoxically - the opposite has been reported frequently enough for thermal pain thresholds to be verified in a meta-analysis [7]. For example, increased thresholds to certain experimental pain modalities have been found in patients with affective disorders [8], [9], [10] and reduced sensitivity for cold pain has been reported in patients suffering from major depression[11]. The neurobiological underpinnings of such findings are not yet understood, but studies have indicated a potential common role of serotonin (5-HT) [12]. Serotonin is involved in a number of homeostatic processes [13], [14]. Importantly, 5-HT modulates nociception both through peripheral and central mechanisms [15] as well as being involved in the regulation of mood [16], [17].Rodents bred for high anxiety display lower sensitivity to thermal pain as compared to those bred for low anxiety and these differences appear to be attenuated by selective serotonin re-uptake inhibitors (SSRIs) [18].

SSRIs target the 5-HT transporter (5-HTT) which is a key player in 5-HT signaling as it terminates the extracellular signal through re-uptake[19]. In humans the promoter region of the gene coding for the 5-HTT (*SLC6A4*) harbors a 43 base-pair insertion/deletion referred to as the 5-HTT linked polymorphic region (5-HTTLPR). This polymorphism consists of a long (l) allele and a short (s) allele. The s-allele occurs with a frequency of between 38% and 57% in various European populations, giving frequencies of s/s-homozygotes ranging between 14% and 29%[20]. The 5-HTTLPR has become one of the most well-studied genetic polymorphisms in psychiatric genetic research[21] and the s-allele, coupled to reduced gene-expression in-vitro [22], has been associated with a number of affective disorders including depression [19], [21]. The promoter region of the *SLC6A4* gene also harbors the single-nucleotide polymorphism (SNP) rs25531 which implies an A to G substitution. The rs25531 has been shown to further alter the degree of 5-HTT gene expression. The minor G-allele is nearly always in phase with the l-allele of the 5-HTTLPR and has been shown to reduce transcriptional efficacy to the level of the s-allele[23]. When studied jointly, as in the present study, the mini-haplotypes constructed from 5-HTTLPR and rs25531 are usually referred to as ‘tri-allelic’ 5-HTTLPR. The fourth allele, S_G_, is very rare and often ignored in studies. Thus, the tri-allelic 5-HTTLPR permits the functional division of individuals into high- (L_A_/L_A_), intermediate- (L_A_/L_G_, S_A_/L_A_) or low- (S_A_/S_A_, L_G_/S_A_) expressors of the 5-HTT [23].

Reports of 5-HTT-knockout mice exhibiting markedly reduced thermal hyperalgesia in a model of neuropathic pain [24], [25] suggest that the human tri-allelic 5-HTTLPR could be an ideal candidate gene for exploring 5-HT related individual differences in thermal pain perception and, possibly, perception of neuropathic pain. Additionally, pharmacogenetic studies, as well as work on 5-HTT knockout animals, suggest that 5-HTT related variability may have a stronger phenotypic impact in females [26], [27]. Furthermore, gender differences in central 5-HT metabolism are seen [28], [29] and the tri-allelic 5-HTTLPR genotype has indeed been shown to interact with gender [30], [31]. Studies aimed at further elucidating such gender by genotype interactions with regard to pain phenotypes may help us understand why chronic pain is more common in women [4] and hopefully lead to improved treatment.

As mentioned, depressed individuals often show elevated thresholds to thermal pain. There is no established mechanistic explanation for this hypoalgesia [7]. Low 5-HTT expression is, however, a known risk factor for depression[32]. Together with the outlined findings in 5-HTT knockout mice, this certainly points to a potential impact of 5-HTT expression on human thermal pain thresholds even in non-depressed individuals. However, there are few studies of the potential influence of 5-HTT expression on experimental pain perception in humans. We therefore investigated how the tri-allelic 5-HTTLPR genotype together with gender may influence thermal pain sensitivity in healthy non-depressed volunteers.

Additionally, we conducted a preliminary investigation of the perception of the so-called thermal grill illusion (TGI) on the background of the studied genotype. The TGI was first described in 1898 by Torsten Thunberg [33]and is a potentially painful sensation that may arise when simultaneously touching juxtapositioned rods of innocuous cold and warm temperatures[34]. Craig and Bushnell have suggested that the illusion depends on the central integration of thermoafferent and nociceptive information with the putative unmasking of burning pain[34]. It has been suggested that the TGI could be of clinical relevance, casting light on mechanisms involved in neuropathic pain [35] including cold-allodynia[36]. The occurrence of cold-allodynia may also be related to the (non-neuropathic) pathological processes involved in chronic wide-spread pain and is, for instance, common in fibromyalgia[37].

Interestingly, there is a large inter-individual variation in the perception of the TGI and as many as one-third of healthy volunteers are reported to be non- or poor-responders to the illusion [36], [38]. As thermal-pain perception appears to be highly influenced by hereditary factors [39], it would therefore be expected that common genetic variants could also account for some of this variability in the TGI. Innocuous thermal information from the skin plays an important part in thermoregulation and related homeostatic processes[40]. As serotonergic mechanisms are involved in such thermoregulation [13], [41] the TGI may provide an interesting complement to the study of noxious thermal pain on the background of differing 5-HTT expression. We therefore separately assessed the perception of pain (i.e. sensory-discriminatory dimension) and unpleasantness (i.e. affective-motivational dimension)[42]of the TGI.

In sum, we tested the hypothesis that the sensitivity to thermal pain as well as the thermal grill illusion (TGI) are associated with the tri-allelic 5-HTTLPR. Healthy volunteers were pre-selected on the basis of gender and genotype, with inferred high or low 5-HTT expression. We hypothesized that low 5-HTT-expressing individuals would exhibit reduced sensitivity to noxious heat and cold as well as be less likely to perceive the TGI less intensely for a given set of cold and warm temperatures. The latter hypothesis follows as an extension of the expected insensitivity to experimental thermal stimuli on the basis of low 5-HTT expression. Furthermore, we expected that any such differences between genotype groups would be more pronounced in females. To the best of our knowledge, this is the first study assessing thermal pain thresholds in relation to tri-allelic 5-HTTLPR. It is also the first attempt at using genetics to account for some of the previously reported inter-individual variability in the perception of the thermal grill illusion.

## Methods

### Participants

The study was approved by the Regional Ethical Review Board in Stockholm (Centrala Etikprövningsnämnden, reference number 2010/716 – 32) and conducted according to the principles expressed in the Declaration of Helsinki. All subjects provided written informed consent and were paid for their participation.

Subjects were selected a-priori based on gender and tri-allelic 5-HTTLPR genotype, with inferred high- (L_A_/L_A_) or low (S_A_/S_A_, S_A_/L_G_) 5-HTT-expression, from a pool of approximately 500 genotyped individuals. Both subjects and experimenters were blinded for the genotype and participants of both genotype groups were included and tested in random order. Subjects in the pool had previously provided a DNA-sample and given informed consent for DNA-analysis and to be contacted for invitation to participate in future experiments.

Individuals in the pool were naïve to our paradigm and had not participated in any previous pain experiments conducted by our group. Importantly, both during recruitment and testing, the nature of the TGI was not revealed– subjects were merely told that the temperatures used throughout the experiment could be painful but not dangerous. To meet the inclusion criteria, participants had to be healthy, non-pregnant, adults without pain problems and not suffer from any present or previous psychiatric disorder. Except for contraceptives, subjects were not included if they took any pharmaceuticals that could potentially interact with pain perception. These criteria were initially assessed by a brief phone interview during the recruitment and also confirmed on the test day.

Forty-four volunteers of European descent were included in the study (see Table 1). Twenty-one subjects were in the low 5-HTT-expressing group (12 females) and 23 individuals were in the high 5-HTT-expressing group (15 females). Two additional subjects partook in parts of the experiment but were excluded due to technical problems (n = 1) or because of reporting current chronic pain problems (despite our pre-screening) during the post-experimental debriefing. The participants in the genotype groups did not differ significantly in age [U =  217.0, z = −0.58, p = 0.57] and women did not differ significantly in menstrual cycle phase [U = 179.0, z = −0.77, p = 0.45] between genotype groups.

**Table 1:** Forty-four healthy participants of European descent were included in the study.

Tri-allelic 5-HTTLPR genotype	N (N females)	Age	Age range	BDI-score	State STAI-score	Trait STAI-score
Low 5-HTT-expression	21 (12)	28.6 yrs +/− 8.4	21 – 54	3.1 +/−3.1	31.3 +/− 7.0	35.2 +/−8.3
High 5-HTT-expression	23 (15)	26.7 yrs +/− 6.2	20 – 52	4.7 +/−5.0	27.5 +/−5.3	35.1 +/−7.3

Participants were selected on the basis of tri-allelic 5-HTTLPR genotype and gender.

### Genotyping

Samples for DNA-extraction were either obtained in the form of 20 ml whole blood or saliva. DNA-extraction from whole blood was performed as described earlier [43] and from saliva using the protocol and reagents in the Oragene® kit (DNA Genotek Inc, Kanata, Canada).

To determine the tri-allelic 5-HTTLPR, PCR reactions were carried out in a total volume of 20 µl containing 50 ng of genomic template, 0.2 nM of each dNTP, 1.0 mM of each primer (Thermo Scientific, Ulm, Germany), 0.05 U/µl Quiagen HotStar®Polymerase, 1 M Q-solution and 1x Buffer. The forward primer sequence was 5′-GGCGTTGCCGCTCTGAATGC-3′ and the reverse 5′-GAGGGACTGAGCTGGACAACCAC-3′. Samples were amplified on a Biorad Tetrade (Biorad, Hercules, CA, USA), following an initial denaturation step for 10 min at 94°C. The amplification consisted of 32 cycles of 30 s denaturation at 95°C, annealing for 30 s at 57°C and elongation for 30 s at 72°C. This was followed by a final elongation for 5 min at 72°C. The described PCR yields long (529 bp) and a short (486 bp) fragment which were visualized with UV, after 2 h separation at 180 V, on a 2.5% Agarose gel gontaining GelRed®. Additionally, 10 µl of the PCR product were digested for 12 h at 37°C with 0.1 µl MSP1 (New England Biolabs, Ipswitch, MA, USA) and 1 µl buffer per sample. The enzyme cuts at a 5′-C/CGG-3′ sequence resulting in fragments from the length of which the tri-allelic 5-HTTLPR genotype can be determined. Thus, L_A_ results in 340 bp, 127 bp and 62 bp; S_ A_ results in 297 bp, 127 bp, and 62 bp; L_G_ results in 174 bp, 166 bp, 127 and 62 bp; S_ g_ (very uncommon) results in 166 bp, 131 bp,127 bp and 62 bp. MSP1-digested PCR products were then visualized using UV-light after being run for 2 h at 180 V on 4% agarose gels containing GelRed®.

### Experimental protocol and methods, an overview

Testing was conducted by the same two experimenters, using ritualized instructions. Upon arrival at the experimental facility, volunteers provided written informed consent. Skin temperature of the ventral forearm was measured bilaterally using an IR-thermometer. Psychophysical testing of perception of the thermal grill illusion (TGI), and its constituent cold and warm temperatures, was conducted in a counterbalanced and randomized order. Each stimulus lasted 20 seconds. Both VAS-ratings of pain (i.e. sensory-discriminatory dimension) and unpleasantness (i.e. affective-motivational dimension) were collected. Subjects then rested for 20 minutes whereupon thermal pain thresholds for cold- and heat-pain were assessed, using the method of limits, over the skin of the right ventral forearm. During the resting period, EMG-data from eye-blinks to non-noxious auditory stimuli was collected as part of a separate experiment (data will be reported elsewhere).

Details are provided below.

### Questionnaires and scales

Two 100 millimeter long visual analogue scales (VAS), printed on the same sheet of paper, were used for subjective ratings of the TGI. One scale captured the sensory experience (‘no pain’ [left]- ‘worst pain imaginable’ [right]) and one the affective dimension [44] (‘not unpleasant’ [left]- ‘the most unpleasant feeling imaginable’ [right]). Subjects were instructed to distinguish between the sensory-discriminative and affective-motivational dimensions of the stimuli. They were shown the two different VAS-scales and told that: “Any feelings of pain and any feelings of unpleasantness of the stimuli should be rated separately. On this scale we want you to rate any feelings of unpleasantness, irrespective of pain. On this scale we want you to rate any feelings of pain, irrespective of unpleasantness.”Subjects completed the state-part of a Swedish version of the State-Trait Anxiety Inventory (STAI) prior to testing. After the entire series of experiments, subjects completed the trait-part of the STAI as well as a Swedish version of Beck Depression Inventory (BDI). Subjects were provided with an envelope for the questionnaires. If any part of a questionnaire was left blank, multiple answers were chosen or answers were ambiguous, the questionnaire was excluded from the analysis.

### Skin temperature measurements

Skin temperature was measured using an infra-red thermometer (Fluke 63, Fluke Sverige AB, Solna, Sweden) over the ventral forearm, bilaterally. An adapter was used to ensure that the distance to the surface of the skin was 5 cm. Similar non-contact procedures of recording skin temperature have been reported to provide accurate measurements [45], [46].

### Thermal grill

#### Apparatus

A custom-made thermal grill was used. The grill consisted of 8 rectangular thin pure-silver plates (80 mm×10 mm×1 mm), housed in a poly-vinyl-chloride unit and spaced 3 mm apart. Silver was chosen due to its extremely high thermal conductivity. The temperature of odd and even numbered silver plates could be controlled separately using circulating water from two baths; one used for cooling and one used for heating water (Julabo models F25-ED and EH-5, VWR International, Stockholm Sweden). A peristaltic pump fitted with two separate pump-heads (Cole-Parmer Model 7553-75, Cole-Parmer Instrument Co, Chicago, USA) circulated the two water pools through the thermode-housing. The water was in direct contact with each plate and entered the housing in separately insulated chambers – allowing juxtapositioned silver bars to achieve different temperatures. A switch allowed the circulation to be set so that odd and even numbered bars either held the same temperature (cold or warm) or alternating cold and warm temperatures (i.e. thermal grill condition).

The temperatures of the water baths were set to levels that gave the desired temperatures at the surface of the silver plates. The system was thus calibrated to achieve 41.0°C–42.0°C (average 41.5°C)and/or 15.0°C–16.0°C (average 15.5°C) at the silver plates. The correct functioning of each element of the thermode was verified prior to each experimental session using a calibrated and highly sensitive surface probe with a sprung thermocouple strip (Testo 925 and probe type-K, calibrated at a SWEDAC-accredited laboratory by Nordtec Instrument AB, Göteborg, Sweden).

##### *Choice of TGI-temperatures*

We had the hypothesis that 5-HTT-groups would differ with regard to thermal-pain threshold but obviously we did not know the potential effect-size of any such difference. To allow a careful dissection of, for instance, pharmacological treatment-effects[38], [47] some previous studies have individualized the temperatures of the TGI in relation to the thermal pain thresholds [36]. Given the preliminary nature of our TGI-investigation we opted to use the more limited approach of one set of warm and cold temperatures. Importantly, it has been suggested that the TGI-percept increases in intensity as a function of the difference between cold and warm temperatures used, rather than in relation to thermal pain thresholds per se[36]. Under the assumption that this is the case - and given the expected association between the studied genotype and with thermal-pain thresholds – such an individualization would in fact have had the potential to introduce a more serious confound than it would have controlled for. To achieve a relatively stable TGI-percept we therefore chose to use a fairly large fixed temperature difference. Although relating their TGI-paradigm to the thermal pain thresholds Bouhassira et al report a maximum temperature differential of 25°C. We therefore chose temperatures usually considered innocuous resulting in a comparable difference (41.5–15.5°C = 26°C).

##### *Testing*

**Figure 1:**
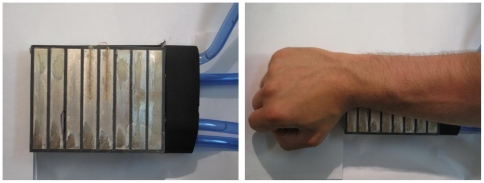
The thermal grill. The thermal grill consisted of 8 individual silver plates housed in a PVC unit. The subjects placed their ventral forearm against the grill's surface, orthogonally to the long axis of the silver plates. Temperatures of odd and even numbered plates were set to 41.0°C–42.0°C and/or 15.0°C–16.0°C.

Subjects were told that the thermode was designed to deliver temperatures which may or may not be painful/unpleasant, but that no temperature would be harmful. The nature of the TGI stimulus was not revealed to subjects who were also blinded to the order of testing. The experimenter used a randomized list to achieve a counterbalanced order between the three conditions, i.e. cold-only, warm-only or cold-and-warm ( = TGI). Before each test the thermode was set to the correct condition; i.e. the thermode elements had achieved the correct temperature prior to skin-contact. Participants were asked to place their ventral forearm over the silver bars a total of three times, 20 seconds each time. The forearm was placed orthogonally to the long axis of the bars (see Figure 1). Immediately after each stimulus, pain (sensory-discriminatory dimension) and unpleasantness (affective-motivational dimension) were rated on two separate VAS-scales (see ‘questionaires and scales’ above). After each 20 second test the subject removed his or her arm from the thermode and a 3 minute inter-stimulus interval ensued. Subjects were asked to use their right arm for the first trial and then alternated arms.

### Thermal pain thresholds

A computer controlled Peltier-type thermode system with a 30 mm×30 mm surface was used for thermal testing (PATHWAY model ATS, Medoc, Israel). Subjects sat comfortably upright in a clinical examination bed with armrests. The thermode with a baseline temperature of 32.0°C was attached to the right ventral forearm using a Velcro-strap. Subjects held a button in their left hand and were instructed to press this at the slightest percept of pain. Subjects were given a verbal cue before each trial and told that they would first receive warm temperatures and then cold. Furthermore, subjects were reassured that the temperatures themselves were not harmful and that it was the percept of pain from the temperature – not temperature itself– that we wanted to test the threshold for.

Baseline was set at 32.0°C, with a change rate of 1.5°C/s and a return rate of 8.0°C/s. An end-to-onset inter-stimulus interval of 30 seconds was used. First 3 heat-pain thresholds were assessed, followed by 3 cold-pain thresholds. For cold-pain testing the program automatically returned the thermode temperature to baseline if a temperature of 0°C occurred before pain had been perceived (i.e. the button pressed). If this happened a threshold of 0°C was assigned to the present and any pending trials.

### Statistics

SPSS Statistics version 17.0 (SPSS Inc, Chicago, USA) was used for all analyses. Data are reported as means ±1 standard error of the mean (SEM). P-values <0.05 were considered significant but Bonferroni-adjusted to control for family-wise error where appropriate as stated. Two-tailed tests were used unless stated (see below). For the questionnaire data, we had an a priori hypothesis that the low-5-HTT-expressing group would show higher ratings of negative affect and therefore used one-tailed tests. Also, based on our a priori hypotheses, we used one-tailed tests for assessing the thermal pain thresholds between the genotype groups.

Shapiro-Wilk tests were used to assess the assumption of normality. Independent-sample t-tests were used to analyze scores from the STAI-questionnaires. To further assess any potential interaction between gender and genotype, univariate analyses of variance, with gender and genotype as fixed factors, were conducted for the trait and state STAI data. This test was also used to assess average skin-temperature.

Non-parametric tests (exact) were used when suitable. Mann-Whitney U tests were used for analysis of age, menstrual cycle phase, BDI-questionnaires and thermal pain thresholds with regard to genotype. Mann-Whitney U tests were also used to follow up gender specific results for the TGI. To validate the TGI paradigm VAS-ratings of cold, warm and TGI were entered into two Friedman's ANOVAs (one for each type of VAS-rating, i.e. sensory or affective) with post-hoc testing using Wilcoxon signed-rank tests. To control for family-wise error during these post-hoc tests, p<0.017 ( = 0.05/3) was considered significant. A Wilcoxon signed-rank test was also used to analyze differences between ratings of ‘unpleasantness‘ versus ‘pain’ for the TGI. Three-way loglinear analyses were used for analyzing categorical data (e.g. ‘cold-pain threshold above 0°C’ or ‘no cold- pain threshold above 0°C ‘) with genotype, gender and pain-category as factors. For breaking down the effects of interactions in these analyses, 2×2 chi-square tests were performed separately for females and males in line with our a-priori hypothesis of gender differences.

## Results

### Questionnaires

See Table 1. One subject's (low 5-HTT-expressing) BDI-questionnaire was excluded. Three subjects in each genotype group had their questionnaires excluded for the state-part of the STAI and a total of three STAI-trait questionnaires were discarded (low 5-HTT-expressing). No significant differences were found between genotype groups for the BDI [U = 205.0, z = - 0.62, p = 0.273], or for the trait-part of the STAI [t(39) = 0.03, p = 0.50]. For state-anxiety, however, the low 5-HTT-expressing group (31.3±1.7) provided significantly higher ratings [t(36)  = 1.91, p = 0.03] compared to the high 5-HTT-expressing group (27.5±1.2). Univariate analyses of variance revealed no interactions between genotype and gender for trait or state STAI-data (both F <1).

### Skin temperature

The mean of the two recordings, one from each ventral forearm prior to any sensory testing, was calculated. The low 5-HTT-expressing group had an average skin temperature of 32.0°C±0.3°C and the high 5-HTT expressing group 32.5°C±0.2°C. Analyses did not reveal a main effect of genotype [F(1, 40) = 1.50, p = 0.23], gender, or their interaction (both F<1).

### Heat-pain thresholds

**Figure 2:**
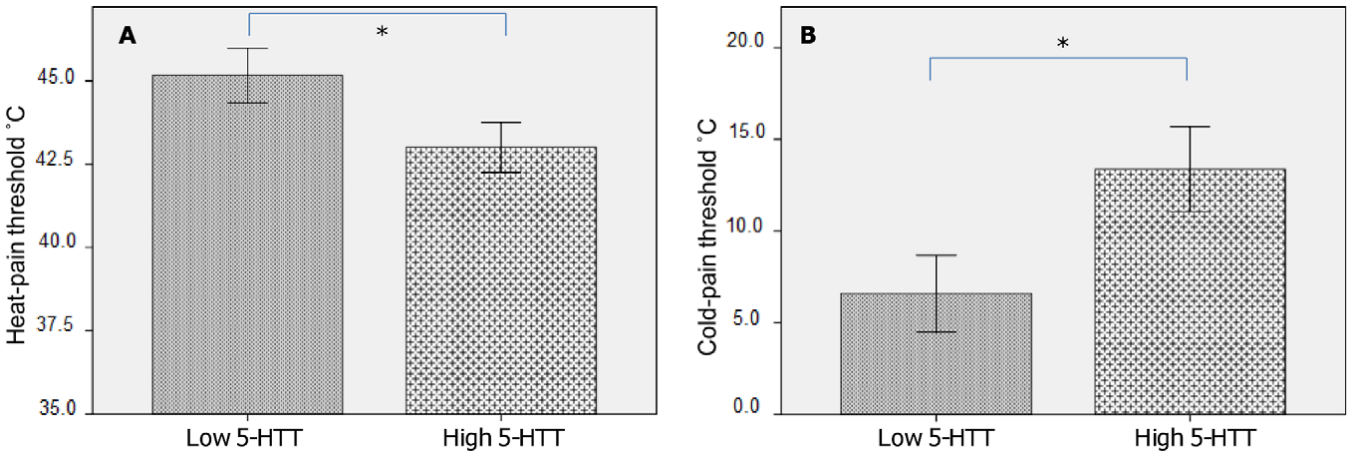
Thermal pain thresholds. A) Heat-pain thresholds. The difference between average heat-pain threshold for the high- versus the low 5-HTT-expressing groups was significant [U = 155.0, z = −2.03, p = 0.02, one-tailed test]. B) Cold-pain thresholds. The difference between average cold-pain threshold for the high versus the low 5-HTT-expressing groups was significant [U = 162.0, z = −1.91, p = 0.03, one-tailed test].

The average threshold temperature for the 3 heat-pain threshold trials was calculated for each subject. The low 5-HTT-expressing group had a mean heat-pain threshold temperature of 45.2°C±0.8°C (median = 45.9°C) compared to 43.0°C±0.7°C (median = 43.9°C) in the high 5-HTT-expressing group. In line with our hypothesis, this difference was significant [U = 155.0, z = -2.03, p = 0.02] (see Figure 2A). To test for interaction effects between genotype and gender, the heat-pain thresholds were then categorized according to whether they were above or below the median heat-pain threshold (see Table 2). A three-way loglinear analysis (genotype x gender x heat-pain category) produced a model that did not show any significant three-way interaction effects [χ^2^(1) = 1.89, p = 0.17].

**Table 2:** Contingency table for heat-pain thresholds.

Gender	Category	Low 5-HTT	High 5-HTT
Male	above median	5	3
	below median	4	5
Female	above median	10	4
	below median	2	11

Number of subjects above or below median temperature for heat-pain threshold.

### Cold-pain thresholds

The low 5-HTT-expressing group had a mean cold-pain threshold of 6.6°C±2.1°C (median  = 1.4°C) compared to 13.4°C±2.3°C (median =  14.2°C) in the high-expressing group. This represented a significant difference between the two genotype groups, in accordance with our a-priori hypothesis [U = 162.0, z = −1.91, p = 0.03] (see Figure 2B). There were clear floor-effects in our data as some subjects did not perceive any pain during the threshold assessment. Therefore, in the second part of this analysis, we divided our sample into 1) subjects who perceived cold-pain and 2) subjects who did not perceive cold-pain (see Table 3). This was done to assess any relation between the genotype groups, gender and category of cold-pain response (i.e. no cold-pain perceived during testing versus cold-pain threshold above 0°C). A three-way loglinear analysis (genotype x gender x cold-pain category) produced a model that retained all effects [likelihood-ratio: χ^2^(0) = 0, p = 1], i.e. the three-way interaction was significant [χ^2^(1) = 4.22, p = 0.04]. To break down this effect, separate chi-square tests were conducted for women and men based on our expectations of gender differences. For women, there was a significantly higher frequency of the low 5-HTT-expressing genotype among individuals that did not perceive cold-pain during testing, as compared to the high 5-HTT-expressing group [χ^2^(1)  = 6.24, p = 0.021]. No such difference was found for men [χ^2^(1)  = 0.08, p<1.00].

**Table 3:** Contingency table for cold-pain thresholds.

Gender	Category	Low 5-HTT	High 5-HTT
Male	cold- pain	7	5
	no cold- pain	2	3
Female	cold- pain	4	12
	no cold- pain	8	3

Number of subjects perceiving cold-pain during testing of thermal thresholds, i.e. reporting pain above 0°C during threshold assessment.

### Correlation between thresholds for heat- and cold-pain

Thermal pain thresholds exhibited a strong and significant non-parametric correlation between heat- and cold-pain sensitivity [Spearman's rho  = −0.61, p<0.001]. I.e. lower sensitivity to cold-pain was associated with lower sensitivity to heat-pain. The correlation remained strong and significant when controlling for both gender and genotype [partial Spearman's rho  = −0.58, p<0.001].

### Thermal grill illusion (TGI)

#### Validation of the TGI-paradigm on the group as a whole

**Figure 3:**
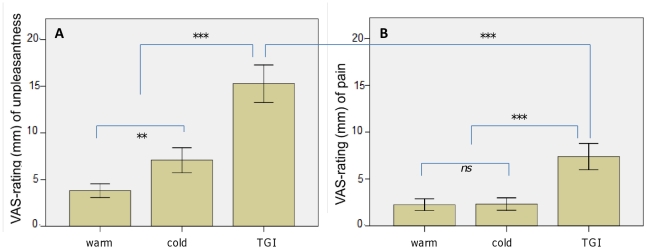
Validation of the thermal grill illusion for affective and sensory dimensions, all subjects. The thermal grill illusion was tested along with its constituent temperatures, in a randomized and counterbalanced order. Subjects provided VAS-ratings of both the affective-motivational (i.e. unpleasantness, see panel A) and sensory-discriminatory (i.e. pain, see panel B) dimensions for each condition. Validation of the thermal grill illusion, for all subjects: ***  =  significant at p<0.001, **  =  significant at p<0.01, ns  =  not significant.

With regard to the VAS-ratings for unpleasantness the cold, warm and TGI conditions differed significantly [χ^2^(2)  = 27.26, p<0.001] (see Figure 3A). Post-hoc testing revealed that TGI obtained a significantly higher unpleasantness-rating than both cold [z = −3.65, p<0.001] and warm [z = −5.00, p<0.001]. Cold achieved higher ratings of unpleasantness than warm [z = −2.70, p = 0.006]. For the VAS-ratings of pain, the three conditions also differed significantly [χ^2^(2)  = 16.90, p<0.001] (see Figure 3B). As expected, post-hoc testing revealed that the TGI was perceived as more painful than its constituent cold [z = −3.39, p<0.001] and warm [z = −4.17, p<0.001] temperatures, with no significant differences in pain ratings between warm and cold [z  = −0.28, p = 0.80]. Comparing the VAS-ratings for pain for the TGI-stimulus (7.4 mm±1.4 mm) with those for unpleasantness (15.3 mm±2.0 mm) showed that the latter was significantly higher [z = −3.76, p<0.001].

#### Correlations between TGI-perception and thermal pain thresholds

Partial non-parametric correlations were calculated between the ratings of pain/unpleasantness and thresholds for heat- and cold-pain, respectively, while controlling for gender and genotype. A higher sensitivity for heat-pain correlated significantly with higher VAS-ratings for unpleasantness [rho = −0.32, p = 0.04] and pain [rho = −0.49, p<0.001] for the TGI. For cold-pain sensitivity a similar pattern emerged for unpleasantness [rho = 0.36, p = 0.02] and just failed to achieve significance for pain [rho = 0.28, p = 0.07].

#### TGI-response, low versus high 5-HTT expression

**Figure 4:**
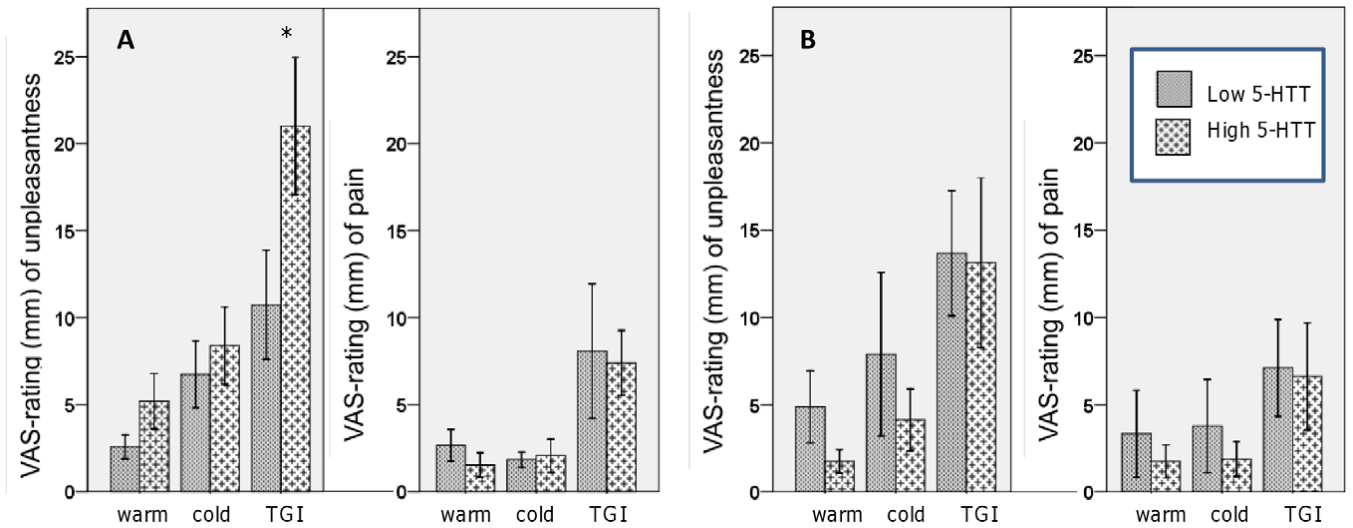
Thermal grill illusion on the basis of tri-allelic 5-HTTLPR genotype and gender. Based on our expectations of gender differences, the genotype groups were further divided into females (panel A) and males (panel B). Females differed significantly in ratings of unpleasantness for the thermal grill, *  =  significant at p<0.05.

As in previous studies [36], [38], some subjects exhibited a poor response to the TGI. A tentative dichotomization into individuals responsive to the TGI versus those with poor-response was therefore conducted. This was done by comparing the VAS-ratings for the cold and warm control conditions with the ratings for the TGI. Both sensory (pain) and affective (unpleasantness) dimensions were considered. We defined a poor TGI-responder as one who either provided the same ratings (i.e. 0 mm) during all conditions for both dimensions (N low 5-HTT  = 5, N high 5-HTT  = 3) or where the ratings for pain and/or unpleasantness were actually lower for the TGI than for the warm or cold temperatures alone. See Table 4. A three-way loglinear analysis (genotype x gender x category of response) produced a model that retained all effects [χ^2^(0)  = 0, p = 1], i.e. the three-way interaction was significant [χ^2^(1)  = 5.64, p = 0.02]. To break down this effect, separate chi-square tests were conducted for women and men. Splitting the analysis by gender consequently gave a highly significant association for women [χ^2^(1)  = 10.71, p = 0.002] but not for men [χ^2^(1)  = 0.052, p<1.00]. That is, there was a significantly higher frequency of low 5-HTT-expressing women in the group with poor TGI-response, compared to the high 5-HTT expressing women. To further follow up on these differences, we explored the differences in ratings of pain and unpleasantness for the TGI between genotype groups split by gender. See Figure 4. Two-tailed tests were used but due to the exploratory nature of the study and constrained sample-size we did not control for multiple comparisons. Women in the high 5-HTT-group provided significantly higher ratings of unpleasantness for the TGI compared to women in the low 5-HTT group [U = 49.5, z = −1.98, p  = 0.047] but not for unpleasantness of cold [U =  84.5, z = −0.27, p = 0.8] or warm [U = 75.5, z = −0.72, p = 0.5].No such differences were seen with regard to pain-ratings [U = 81, z = −0.44, p  = 0.68].

**Table 4:** Contingency table for perception of the thermal grill illusion (TGI).

Gender	Category	Low 5-HTT	High 5-HTT
Male	TGI-responder	5	4
	Poor-responder	4	4
Female	TGI- responder	2	12
	Poor-responder	10	3

VAS-ratings of the sensory-discriminatory dimension (pain intensity) and the affective-motivational dimension (unpleasantness) were used in this tentative dichotomization of subjects. See section ‘TGI-response, sensory vs affective ratings’ for the criteria used.

## Discussion

Our main finding was that thresholds to thermal pain sensitivity are associated with the tri-allelic 5-HTTLPR. As hypothesized, the low 5-HTT-expressing group exhibited significantly reduced sensitivity to heat- and cold- pain when compared to the high 5-HTT-expressing group. Furthermore, an interesting genotype by gender interaction emerged in that there was a significantly higher frequency of women in the low 5-HTT-expressing group, compared to women in the high-expressing group, who did not perceive cold-pain at or above 0°C. The thresholds for heat and cold-pain were strongly correlated, remaining so while controlling for gender and genotype. On this background, the perception of the thermal grill illusion (TGI) was shown to correlate to such thresholds. Additionally, females in the low 5-HTT expressing group showed a relatively poor response to the TGI, providing lower ratings of unpleasantness when compared to females in the high 5-HTT expressing group.

To the best of our knowledge, our results are the first report linking the tri-allelic 5-HTTLPR to thermal pain thresholds. However, a recent publication reports of no relationship between the (bi-allelic) 5-HTTLPR and heat-pain thresholds[48]. These divergent results may be explained by differences in experimental paradigms as well as statistical power. Our pain threshold measurements were based on a rate of temperature increase of 1.5°C/s; reference values from 300 healthy subjects were recently reported in a study employing 1.5C°/s change rate[49]. Potvin et al employed a more gradual increase of 0.3°C/s possibly exposing the measure to effects of temporal summation [50]. Also, the previous study employed a bi-allelic genotyping approach, without consideration of the rs25531. There is evidence that using the tri-allelic mini-haplotype confers additional resolution as the rs25531 G-allele, on the background of the long-allele of 5-HTTLPR, reduces the transcriptional efficacy to the level of the short-allele[23] and thereby better captures the functional variation in the 5-HTT promoter.

Any association between a genetic polymorphism and a pain-phenotype begs the question whether the putative effects of the polymorphism are due to peripheral or central effects, or a combination of the two. The majority of 5-HT is located outside of the central nervous system[51] and it is important to acknowledge the possibility of peripheral differences, associated with the tri-allelic 5-HTTLPR, in interpreting our present results. Increased plasma 5-HT has been reported in complex regional pain syndrome 1 (CRPS1) [52], a key feature of which is unrelenting burning pain. The 5-HTT mediates the uptake of 5-HT into platelets and low expression has been coupled to increases in peripherally circulating 5-HT [51], [53]. In animal models 5-HT has been shown to sensitize unmyelinated primary C-fiber afferents [54]. Also, as the name implies, serotonin is a vasoactive molecule and as such could affect surface skin temperature and, through effects secondary to this, thermal pain perception. However, no such significant differences in skin temperatures were found on the basis of genotype.

Our results may equally be influenced by differences at the spinal level as descending serotonergic projections from the brainstem are highly involved in the inhibition [55] as well as facilitation of nociceptive information[56].Primary afferents carrying both noxious and innocuous thermal information synapse in lamina I which is thus implicated in normal thermosensitivity, thermal pain perception and – as suggested by Craig- the TGI [57]. Importantly, lamina I receives the highest density of such descending 5-HT innervations [58] and differential 5-HTT expression is known to alter the functional 5-HT receptor availability in rodents [59]. With regard to supraspinal mechanism imaging has revealed an insular response to both noxious and innocuous heat and cold [60], [61]. This fits well with the fact that lamina I afferents are relayed to the insula, believed to be highly involved in homeostatic processes and interoception[57]. Craig and co-worker's positron tomography emission (PET) - imaging of the TGI also revealed activation of anterior cingulate cortex (ACC). Whereas noxious thermal stimuli were also seen to engage the ACC, the constituent temperatures of the TGI activated only the insula but not the ACC [61]. The ACC is suggested to be involved in the immediate appraisal of pain unpleasantness [42] and in relation to 5-HTT, PET- imaging has revealed differences in the metabolic activity in the ACC on the basis of 5-HTTLPR[62]. Additionally, a functional-MRI study has shown the insula and ACC to be affected by subchronic administration of SSRI:s during processing of affective stimuli [63]. The authors suggest that SSRIs may modulate the anticipation of aversive stimuli through the dampening of activity in these regions.

We found that the cold-pain thresholds and heat-pain thresholds were strongly and significantly correlated (partial rho  = −0.58, p<0.001). Given the somewhat different mechanisms in peripheral transduction between the two types of noxious stimuli [64], this is not a trivial finding. Previous studies have demonstrated large inter-individual differences in thresholds for cold-pain perception [49], [64], [65], findings which our results corroborate and may contribute to an understanding of. Surprisingly few studies have addressed the actual correlation between noxious cold and noxious heat thresholds in healthy subjects. One study reported a correlation coefficient of 0.34 [66] and another 0.23 [67]. Of great importance in this regard are the studies on inbred mouse-strains conducted by Mogil and colleagues. The group showed that hot and cold nociception are strongly genetically correlated in mice (r =  0.49–0.77) [39], indicating that physiological mechanisms common to both traits share genetic underpinnings. Further, using temperatures in the innocuous-range in healthy volunteers, Green and Akirav report strong correlations of perceived intensity of cold with perceived intensity of warmth (r = 0.83) [68]. Importantly, the study controlled for possible inter-individual peripheral differences in innervations density and spatial summation, as well as for psychological factors influencing the actual rating procedure. As suggested by the authors, such results may reflect that the intensity of a thermal percept is subject to strong central modulation, possibly in relation to thermoregulation. Importantly, for the interpretation of the present results 5-HT neurons of the medullary raphé are involved in neural pathways subserving homeostatic and thermoregulatory processes [13], [41], [69].

The TGI uses innocuous temperatures and is therefore interesting in relation to such putative thermoregulatory processes. Based on neurophysiological recordings of spinothalamic neurons in anesthetized cats, Craig and Bushnell suggested a model of central dishinhibition for explaining the TGI. Recordings were made in lamina I spinothalamic tract neurons from nociceptive specific cells (NS), thermoreceptive cells responsive to cooling (COLD) and multimodal cells responsive to noxious heat as well as pinch and cold (HPC). Whereas the NS cells remained unaffected by cold and warm as well as the two interlaced (i.e. TGI-condition), COLD cells were potently inhibited whereas the HPC were inhibited to a much lesser degree by the thermal grill stimuli. The suggested thermosensory inhibition model thus posits the central inhibition of burning pain by cold, which is disrupted during the TGI-stimuli [34]. Based on elaborate psychophysical investigations, Bouhassira and co-workers suggest a somewhat different model involving a simple ‘addition’ between COLD and HPC activity[36]. Importantly, both models involve a supraspinal integration of COLD with multimodal (HPC) afferent activity.

Whereas several studies report a perception of ‘synthetic heat’ rather than actual pain from the TGI [70], our results support reports from Craig and Bushnell [10] as well as Bouhassira and co-workers [36] that the TGI may indeed be perceived as painful. However, the ratings of pain – although significantly higher for the TGI than control conditions – were indeed notably low. Interestingly, the VAS-ratings of the affective-motivational dimension (upleasantness) were significantly higher than those for the sensory-discriminatory dimension (pain). Being more of an ‘illusion of unpleasantness’ rather than of pain does not necessarily reduce the TGI's potential value as a model for pain research in humans, however. For example, in patients with spinal cord injury involving the spinothalamic tract, dysesthesias of burning quality are frequently reported and regardless of whether they are described as painful or not, such burning dysesthesias may be functionally limiting [71].

Using a fixed-temperature paradigm for cold and warm we demonstrate how the intensity of sensory and affective dimensions of the TGI correlate with sensitivity to thermal pain. On this background, a tentative set of poor-responders for the TGI-emerged, i.e. low 5-HTT expressing females who rated the TGI as less unpleasant compared to high-expressing females. The interpretation of these TGI-results, however, is limited by the demonstrated association between thermal pain thresholds and genotype/gender. Such potential confounds are partially mitigated by the conclusions of Bouhassira and co-workers that the strength of the TGI-percept relates “to the magnitude of the differential of the combination of cold-warm temperatures, but not to their proximity to the thermal pain thresholds”[36]. However, given the preliminary nature of our study we only tested one set of cold and warm temperatures and are therefore unable to corroborate these results. In our study cold and heat pain thresholds were correlated both to each other as well as to the sensory and affective dimensions of the TGI. Accordingly, our results are entirely congruent with a ‘general integrative model’ of the TGI based on differential activity between COLD and HPC [36]. It is therefore unlikely that our subjects would have perceived the TGI more intensely for a smaller gap between cold and warm temperatures and indeed we tested the TGI using a fairly large such gap between cold and warm temperatures. Experiments where TGI-temperatures have been individualized report a differential of up to 25°C [36] (Boettger et al [72] report differences averaging up to 26.8°C, but used a gap averaging 22.8° for their actual experimental manipulation, see below). As mentioned, this was a rationale for choosing the fixed temperatures used in our experiment (i.e. 15.5°C and 41.5°C) compared to, for instance, those used by Craig and co-workers (20°C and 40°C)[61].

Females in the low 5-HTT-expressing group had markedly reduced sensitivity to threshold cold-pain. These females also provided lower ratings of TGI-unpleasantness compared to those in the high 5-HTT-expressing group. Probing potentially separate mechanisms, the recent report from Boettger and colleagues is interesting [72].The effect of sad-mood induction on the perception of the TGI was studied in females. The sad-mood was shown to intensify the perception of the TGI without significantly influencing thermal pain thresholds. Such results parallel reports of sad-mood induction on the ratings of unpleasantness of tonic noxious heat [73]. Genetically inferred low 5-HTT-expression has been demonstrated to correlate with an increased reactivity to negative environmental cues and is indeed likely to increase the propensity of having a sad-mood induced [74], [75], [76], [77]. We therefore find it likely that the mechanisms involved in the effects of sad-mood induction on TGI-perception are different from those underlying our findings. We speculate that the associations between genotype/gender and perception seen in our study stem from differential activity in the early-stages of the central processing and putative integration of thermoafferent and nociceptive information. Our results therefore complement studies involving such manipulations of the perception of the TGI. The low 5-HTT expressing group may actually be particularly relevant to study in the setting of sad-mood induction but, as suggested by our results, healthy non-depressed females in this group are likely to exhibit a lower baseline perception of affective-motivational dimension of the TGI.

Strigo and co-workers cast light on some of the supraspinal mechanisms related to pain perception in depressed patients [78].The authors studied affective bias, indexed by dividing ratings of unpleasantness by ratings of pain for a given thermal stimuli. Depressed patients had an increased affective-bias compared to non-depressed controls and this bias was most apparent in the innocuous range of warm temperatures. The authors suggest the term ‘emotional allodynia’ to describe the phenomenon of “abnormal elicitation by subthreshold stimuli of the affective-motivational component associated with the perception of pain.” In the present study we demonstrate that the TGI is more unpleasant than painful. Additionally, we demonstrate how the affective dimension may be selectively dissociated from the sensory dimension on the basis of putative serotonergic mechanisms and gender. The TGI could therefore be interesting to the study of such ‘emotional allodynia’, especially as immediate pain intensity and unpleasantness partially may depend on different spinothalamic tract neurons. It has been suggested that fibers ascending to the medial thamaic nuclei (i.e. the posterior part of the ventral medial nucleus - VMPo and the ventral caudal part of the medial dorsal nucleus - MDvc) contribute more directly to the affective dimension of potentially painful percepts, compared to those ascending to the ventral posterior lateral thalamic nucleus (VPL)[42]. Thus, hypothetically, the dissociation between pain and unpleasantness in the TGI-percept may be due to differential responses to thermoafferent stimuli in these two different ascending pathways.

The present study was partially inspired by the fact that patients with depression are frequently reported to exhibit elevated thresholds to thermal pain, as discussed in the meta-analysis by Dickens and colleagues[7].Somewhat paradoxically, such patients also have an increased risk of develop chronic pain-pathologies [2]. Paralleling these findings, reduced 5-HTT expression has been reported to be associated with an increased risk of developing chronic musculoskeletal pain[79], [80]. Our results may thus suggest a common 5-HTT related mechanism of how hypoalgesia to transient threshold thermal stimuli may be observed in individuals with a putatively increased risk of developing pathological. Given the nature of the present experiment, the result of a slightly higher state-anxiety in the low 5-HTT-expressing group, compared to the high-expressing group, was expected. The design of our experiment does not permit a definite untangling of the association of tri-allelic 5-HTTLPR on pain and anxiety. Our participants were nonetheless healthy individuals without a self-reported history of affective disorders and did not differ in trait anxiety or in depression-score and, importantly, no interaction with gender was seen for the state-anxiety.

Other studies have compared pain-relevant anxiety with the focus of attention[81], [82] and illustrate the complicated relationship between laboratory induced anxiety and sensitivity to pain[83].Strigo and co-workers report of an increased reactivity in the anterior insular region, ACC as well as amygdala in depressed individuals during the anticipation of painful heat [84]. Speculatively, such cortico-limbic reactivity may be more enhanced in the modulation of tonic suprathreshold noxious stimuli as compared to transient threshold pain and may therefore help to explain the propensity of depressed individuals to develop chronic pain. This reasoning is partially compatible with the interpretation suggested by Dickens and colleagues. The authors suggest that impairments in attention to relatively mild environmental stimuli may underlie hypoalgesia in depressed subjects at or around pain-perception thresholds. They also speculate that such effects would be less for higher noxious intensities [7].

Differences in a number of receptor-systems may be involved in the demonstrated association between tri-allelic 5-HTTLPR and thermal pain thresholds. We recently reported that the tri-allelic 5-HTTLPR is associated with the response of the short acting opioid remifentanil and suggested that differences in the functional regulation of 5-HT_1A_ receptors may be involved[85]. 5-HT_1A_ receptors are known to be functionally down-regulated in 5-HTT-knockout animals[86] and, contrary to their effects during tonic pain, 5-HT_1A_ receptors are reported to exhibit pro-nociceptive properties during phasic stimulation [87]. This explanation would fit well with our present results in terms of the putatively down-regulated 5-HT_1A_ receptors in the less thermo-sensitive low 5-HTT-expressing group. It would also suggest a molecular mechanism where hypoalgesia to phasic threshold stimuli could be observed in individuals who still might be prone to develop pathological pain[85].

The observed interaction of gender with the tri-allelic 5-HTTLPR genotype in relation to pain-phenotype is in line with numerous previous findings of gender differences with regard to 5-HT-related biology. Results from PET-imaging indicate that the synthesis of 5-HT appears to be greater in males than females, with the surprisingly high magnitude of 50% [88]. In particular, there are reports of an interaction between gender and 5-HTTLPR genotype [89] and a recent PET-study showed that tri-allelic 5-HTTLPR genotype affects the binding to central 5-HT_1A_ receptors in women but did not reveal any such association in men [90]. On the behavioral side, gender has been reported to modulate the genotype effect in depression [91], central fatigue [30], as well as stress reactivity [92]. These findings are further supported by available animal data. In male mice, both estrogen and testosterone have been shown to influence 5-HTT expression [93]. Further, female 5-HTT knockout mice show dramatically increased 5-HT synthesis compared to male knockouts [94].

A study of particular interest for the interpretation of our present results showed electrophysiological differences in neurons of the dorsal raphé, between 5-HTT-knockout mice and controls, during application of a 5-HT_1A_ agonist. The recorded differences were especially pronounced in female animals [27]. In relation to thermoregulation, 5-HT_1A_ receptor activation has been shown to be affected by gonadal hormones [95]. Importantly, ovariectomized rats have been shown to decrease the density of spinal 5-HT_1A_ receptors whereas injections of estrogen induce 5-HT_1A_ receptor expression in the superficial lamina [96], [97]. It is also possible that our results could be explained by other receptor systems – interacting with 5-HT. Of particular interest in relation to both thermal pain thresholds and paradoxical burning, such as that caused by the TGI, is the neurokinin 1 (NK_1_) receptor. The NK_1_-receptor binds substance P which mediates the burning sensation of e.g. capsaicin [98], [99]. Studies in rats reveal that descending serotonergic neurons synapse preferentially to lamina I projections neurons expressing the NK_1_-receptor [100] and a sexually dimorphic regulation of this receptor-system may occur [101], [102].

Several important limitations of our study should be noted. The sample size of our study was fairly small and the results need to be interpreted with the proper caution. High and low 5-HTT-expression was inferred by genotype, rather than measured directly and, as in all genetic association studies, causality cannot be directly assessed. Our result are limited to individuals of European descent and allele frequencies of 5-HTTLPR are known to vary substantially throughout ethnic groups [20]. The interpretation of our TGI-results should take into account the demonstrated association of thermal pain thresholds with the tri-allelic 5-HTTLPR. Future studies relating 5-HTT expression to the perception of thermal grill may therefore benefit by including several combinations of fixed warm and cold temperatures as well as testing temperatures individualized according to cold- and heat-pain thresholds.

In sum, we demonstrate a strong association between sensitivity for detecting thermal pain and tri-allelic 5-HTTLPR. Low 5-HTT expression, inferred by genotype, was associated with a relative hypoalgesia to phasic thermal pain. Our results also show that gender interacts with genotype for perception of cold-pain such that women in the low-5HTT expressing group are less sensitive. Overall, the TGI percept was found to lie more along the affective-motivational domain (i.e. unpleasantness) than sensory-discriminatory (i.e. pain). The results for the TGI were congruent with those for thermal pain. Taken together with the highly significant correlation between cold- and heat-pain thresholds, this suggests a strong influence of central modulation. Therefore, although peripheral effects may also be involved in the present findings, we suggest that the available evidence also points to a role of the differential regulation of both noxious and innocuous thermal information along the neuraxis, on the basis of tri-allelic 5-HTTLPR and gender. Despite the fact that low 5-HTT expression is a risk-factor for chronic pain we found this to be associated with hypoalgesia to threshold-level thermal stimuli. Depression is, however, also associated with low 5-HTT expression and depressed patients often have a reduced sensitivity to thermal pain. Our results point to mechanisms that may be involved in explaining such paradoxical hypoalgesia. A better understanding of the molecular underpinnings of these phenomena may prove important in improving treatment options for pathological pain. As this study further illustrates, the thermal grill may provide a valuable tool in exploring the affective-motivational dimensions of such putative mechanisms.
